# Wall Paper Poison

**Published:** 1874-02

**Authors:** 


### WALL PAPER POISON.

Almost all green wall paper is poisonous, as arsenic is employed to produce that color. Some red colors have also a dangerous amount of arsenic. It is wisest to ascertain before paper is put on a wall whether it contains a poisonous ingredient. Soak a piece of the paper in a concentrated solution of sodium nitrate, and equal parts of water and alcohol—that is add the sodium until the liquid, dissolves no more of it. Burn the paper in a saucer. It smoulders rather than flames. Pour water over the ashes boil and filter. The filtrate is acidified with dilute sulphuric acid, and permanganate of potash is added slowly as long as the red color disappears or changes to yellow brown upon warming, and finally a slight excess of chameleon solution is present. If the liquid becomes turbid it is to be filtered. After cooling, more dilute sulphuric acid is added, and also a piece of pure clean zinc, and the flask closed with a cork split in two places. In one split of the cork a piece of paper moistened in silver nitrate is fastened, in the other a strip of parchment paper dipped in sugar of lead.

If arsenic is present the silver soon blackens. The lead paper is merely a check on presence of sulphuric acid.

